# Development of chitin nanofiber coatings for prolonging shelf life and inhibiting bacterial growth on fresh cucumbers

**DOI:** 10.1038/s41598-023-39739-6

**Published:** 2023-08-14

**Authors:** Supachok Tanpichai, Laphaslada Pumpuang, Yanee Srimarut, Weerapong Woraprayote, Yuwares Malila

**Affiliations:** 1 Learning Institute, King Mongkut’s University of Technology Thonburi, Bangkok, 10140 Thailand; 2 Cellulose and Bio-Based Nanomaterials Research Group, King Mongkut’s University of Technology Thonburi, Bangkok, 10140 Thailand; 3 National Center for Genetic Engineering and Biotechnology (BIOTEC), National Science and Technology Development Agency (NSTDA), Pathum Thani, 12120 Thailand; 4 International Joint Research Center On Food Security (IJC-FOODSEC), Thailand Science Park, Pathum Thani, 12120 Thailand

**Keywords:** Biochemistry, Chemistry, Materials science

## Abstract

The widespread usage of petroleum-based polymers as single-use packaging has had harmful effects on the environment. Herein, we developed sustainable chitin nanofiber (ChNF) coatings that prolong the shelf life of fresh cucumbers and delay the growth of pathogenic bacteria on their surfaces. ChNFs with varying degrees of acetylation were successfully prepared via deacetylation using NaOH with treatment times of 0–480 min and defibrillated using mechanical blending. With longer deacetylation reaction times, more acetamido groups (–NHCOCH_3_) in chitin molecules were converted to amino groups (–NH_2_), which imparted antibacterial properties to the ChNFs. The ChNF morphologies were affected by deacetylation reaction time. ChNFs deacetylated for 240 min had an average width of 9.0 nm and lengths of up to several μm, whereas rod-like structured ChNFs with a mean width of 7.3 nm and an average length of 222.3 nm were obtained with the reaction time of 480 min. Furthermore, we demonstrated a standalone ChNF coating to extend the shelf life of cucumbers. In comparison to the rod-like structured ChNFs, the 120 and 240-min deacetylated ChNFs exhibited a fibril-like structure, which considerably retarded the moisture loss of cucumbers and the growth rate of bacteria on their outer surfaces during storage. Cucumbers coated with these 120 and 240-min deacetylated ChNFs demonstrated a lower weight loss rate of ⁓ 3.9% day^−1^ compared to the uncoated cucumbers, which exhibited a weight loss rate of 4.6% day^−1^. This protective effect provided by these renewable ChNFs holds promising potential to reduce food waste and the use of petroleum-based packaging materials.

## Introduction

Food packaging, which is generally made from petroleum-based polymers, such as polyethylene (PE), polypropylene (PP), and poly(ethylene terephthalate) (PET), plays an important role in protecting food from external physical, microbiological, and chemical damage^1–3^. Consequently, food quality and freshness are preserved, and food waste is reduced^4,5^. Owing to these benefits, which have become more essential in light of the COVID-19 pandemic, it is estimated that the global food packaging industry will be worth 464 billion USD by 2027^6,7^. The high consumption of fossil-based packaging and its time-consuming degradation kinetics have negatively affected the environment and wildlife in the form of landfill waste, greenhouse gas emissions, and microplastics^4,8–10^. Therefore, the development of environmentally friendly and biodegradable packaging materials has attracted considerable attention as a practical alternative^6,9,11–13^. Biopolymers, such as polysaccharides, lipids, and proteins, are promising materials in packaging sectors owing to their biodegradability, biocompatibility, and nontoxicity^3,7,9^.

Chitin (poly(*β*-(1-4)-*N*-acetyl-d-glucosamine)) is the second most abundant biopolymer on Earth after cellulose^14–16^ and is of considerable interest owing to its chemical stability, biocompatibility, biodegradability, nontoxicity, and mechanical properties^17,18^. Chitin is a semicrystalline polymer with a microfibrillar architecture embedded in a protein matrix found in the exoskeletons of arthropods, including shrimps, crabs, and lobsters^10,14,15,19^. Each chitin microfibril comprises nanofibers with a width range of 2–5 nm and lengths of up to several μm^20–22^. Chitin nanofibers (ChNFs) exhibit superior performance with a Young’s modulus of ⁓ 40 GPa, strength of 1.6 GPa, and density of 1–1.3 kg m^−3^. Moreover, ChNF films have been reported to have much better barrier properties (O_2_ and CO_2_) than commercial PP, PE, and PET films owing to the highly crystalline structure of ChNFs^23–25^. Owing to these outstanding properties associated with biodegradability and sustainability, ChNFs have been widely used in various applications, such as nanocomposites, membranes, drugs, coating, and functional foods^2,20,22,26^.

Crab and shrimp shells are generally treated as food waste in the seafood industry^10,27^. Thailand has a large quantity of shrimp shell waste which is potentially used as a raw material to prepare ChNFs^28^. To disintegrate individualized ChNFs, the high mechanical force generated by a powerful machine (i.e., high-pressure homogenization and grinding) is necessary to destroy the strong hydrogen bonding between nanofibers^22,29^. However, this mechanical processing consumes a lot of energy, incurring high cost^29,30^. Ifuku et al*.*^19^ found that although ChNFs were successfully prepared from crab shells using an industrial grinder, the width distribution of ChNFs was in a wide range of 10–100 nm. Similarly, successful individualization of ChNFs could not be achieved using only ultrasonication^31^. Therefore, chemical modification of the chitin surface has been performed to yield individualized ChNFs^27^. Primary hydroxyl groups at the C6 position in chitin molecules were selectively converted to carboxylate groups via 2,2,6,6-tetramethylpiperidine-1-oxyl radical (TEMPO)-mediated oxidation. The chitin carboxylate content was found to dominate the electrical repulsion between nanofibers with anionic charges, resulting in the individualization of ChNFs via mechanical treatment^27^. More individualized ChNFs were obtained from chitin with higher carboxylate content^27^. However, the high chemical expense of TEMPO limits the widespread use of this chemical route. Deacetylation by NaOH to replace acetamido groups by amino groups in chitin molecules has been also reported to weaken hydrogen interaction between chitin molecular chains, assisting individualization of ChNFs^30,32^. Machida et al*.*^32^ reported that ChNFs with smaller widths could be obtained with the higher conversion of acetamido groups to amino groups via deacetylation by NaOH. It was also suggested that these amino groups are important for antimicrobial properties via the interaction between protonated amino groups and negative residues on bacterial cell membranes^33,34^. Therefore, tailoring the number of amino groups on the ChNF surface via deacetylation by NaOH would be an efficient method to obtain individualized nanofibers associated with antimicrobial properties.

Although ChNFs have been prepared from the exoskeletons of crustaceans and cell walls of fungi via various chemical treatments^3,32,35,36^, the effects of deacetylation reaction time on the properties of ChNFs have not been extensively studied, and the efficiency of ChNFs as a coating for food-contact packaging applications remains unknown. Herein, we report properties of ChNFs prepared from shrimp shell waste with various deacetylation reaction times. The effect of the deacetylation reaction time on the characteristics of ChNFs, including chemical structure, degree of acetylation (DA), crystallinity, thermal properties, zeta (ζ)-potential, morphology, and antimicrobial properties, was evaluated. Then, the ChNF suspensions were applied as standalone coatings to extend the shelf life of fresh cucumbers, and the moisture loss and microbial activities of the ChNF-coated cucumbers were investigated for the first time. Given its promising performance, the sustainable and biodegradable ChNF coating shows great potential for postharvest and food packaging applications, which is an effective approach to reducing food waste and the consumption of single-use plastic packaging.

## Materials and methods

### Materials

Dried shrimp shells (*Litopenaeus vannamei*) were purchased from Marine Bio Resource Co., Ltd. (Thailand). Concentrated HCl (6 N) and ethanol (99.5%) were supplied by Fujifilm Wako Pure Chemical Industries (Japan). NaOH pellets (97%) were provided by Nacalai Tesque Inc. (Japan). Fresh cucumbers (*Cucumis sativus*) used in this study were purchased from a local supermarket in Bangkok, Thailand. Cucumbers with consistent shapes and colors and without any signs of fungal infection or damage were carefully selected for experiments. The experiments involving cucumbers were conducted in accordance with relevant institutional, national, and international guidelines and legislation.

### Preparation of chitin nanofibers (ChNFs)

To extract ChNFs from shrimp exoskeletons, a series of chemical treatment steps were applied^20,27,37,38^. Shrimp shells were initially crushed into powders using a blender. The shrimp shell powders (80 g) were demineralized in 1200 mL 2 M HCl, vigorously stirred for 4 h at room temperature, and washed with distilled water until neutral. The treated powders were subsequently soaked in 1000 mL ethanol with continuous stirring for 48 h at room temperature to eliminate pigments and then washed with distilled water several times. Finally, the resultant chitin powders were deacetylated with 30% NaOH at 90 °C for specific treatment times of 120, 240, and 480 min, neutralized, and further dried at 60 °C for 24 h. Subsequently, the deacetylated chitin powders were diluted with distilled water, and a few drops of acetic acid were introduced into the 0.75 wt% chitin suspension to obtain a pH of ⁓ 3. Chitin was defibrillated using high-speed blending (Stormmix blender 3500W, Thailand) at 42,000 rpm for 7.5 min. This mechanical disintegration was repeated four times in 15 min intervals to avoid overheating. The ChNF suspensions were kept at ⁓ 4 °C before use. The ChNF samples deacetylated for 0, 120, 240, and 480 min were denoted as C0, C120, C240, and C480, respectively.

### Fourier-transform infrared (FTIR) spectroscopy

The FTIR spectra of the ChNF samples were recorded using a Nicolet iS5 FTIR spectrometer (Thermo Fisher Scientific, USA) with an attenuated total reflectance mode. The ChNF materials were analyzed in the wavenumber region of 600–4000 cm^−1^ at a resolution of 4 cm^−1^ with 32 repeated scans.

### Solid-state ^13^C nuclear magnetic resonance (NMR) spectroscopy

Structural analyses of the deacetylated ChNF samples were performed using solid-state ^13^C NMR spectroscopy (Bruker Avance III HD/Ascend 400 WB, USA). The NMR spectra were recorded at room temperature using a ^13^C frequency of 100.6 MHz and accumulation of 4096 scans. The DAs of the ChNF materials were determined from the integral (*I*) of the methyl group (CH_3_) and C atoms (C_1–6_) of the chitin backbone structure using the following equation^39,40^:

1 $$\mathrm{DA}\, \left(\%\right)=\frac{{I}_{{\mathrm{CH}}_{3}}}{\left({I}_{{\mathrm{C}}_{1}}+{I}_{{\mathrm{C}}_{2}}+{I}_{{\mathrm{C}}_{3}}+{I}_{{\mathrm{C}}_{4}}+{I}_{{\mathrm{C}}_{5}}+{I}_{{\mathrm{C}}_{6}}\right)/6}\times 100$$

### X-ray diffraction (XRD)

XRD of the deacetylated ChNF samples was performed using an X-ray diffractometer (D8 DISCOVERY, Bruker AXS, Germany). The ChNF samples were scanned in the 2*θ* range of 5–50° using Cu Kα radiation (wavelength of 1.54 Å) generated at an acceleration voltage of 40 kV, current of 40 mA, step increase of 0.02°, and step speed of 0.8 s. The crystallinity degrees of the ChNF samples were calculated as a function of the deacetylation treatment time from the intensity of the diffraction peak located at 19.6° (*I*_110_), corresponding to the crystalline region, and the amorphous area obtained from the baseline intensity located at 16.0° (*I*_AM_) using the following equation^27,41^:

2 $$\mathrm{Crystallinity}\, \left(\%\right)=\left(\frac{{I}_{110}-{I}_{\mathrm{AM}}}{{I}_{110}}\right)\times 100$$

### Thermogravimetric (TG) analysis

The thermal stabilities of the ChNF samples were determined using a Pyris 1 TG analyzer (PerkinElmer, USA). A ChNF sample with a weight of ⁓ 10 mg was held at 110 °C for 20 min to remove moisture and then heated to 800 °C at a heating rate of 10 °C min^−1^ under a N_2_ flow rate of 50 mL min^−1^.

### ζ-Potential measurement

The surface charges (ζ-potential) of the ChNF suspensions (0.1 wt%) were determined using dynamic light scattering (Zetasizer, Nano ZSP, Malvern Panalytical Ltd., UK) at 25 °C.

### Transmission electron microscopy (TEM)

The morphologies of the deacetylated ChNFs were monitored using TEM (JEM-1400, JEOL, Japan) operating at an acceleration voltage of 80 kV. A drop of 0.01 wt% ChNF suspension was deposited on a copper grid and stained with 1% uranyl acetate for 3 min. The average widths and lengths of the deacetylated ChNFs were determined by measuring 100 individual nanofibers using the ImageJ software.

### Application of ChNFs for the shelf life extension of fresh cucumbers

Fresh cucumbers were initially washed with tap water to remove dirt and patted dry with a paper towel. Five cucumbers were then coated with each 0.75 wt% ChNF suspension (C0, C120, C240, and C480) via immersion in the suspension for 2 min. The coated cucumbers were air-dried at room temperature for 5 min. The coating process was repeated four more times for each cucumber to obtain uniform and complete coating. Cucumbers coated with distilled water using the same procedure were used as a control sample. The coated cucumbers were then stored at the temperature of 30 ± 3 °C for 5 days to assess the impact of the ChNF coatings on the extension of their shelf life. The weight and appearance of the cucumbers were monitored daily.

### Antibacterial activity and bacterial inhibition on chilled cucumbers

**Figure 1:**
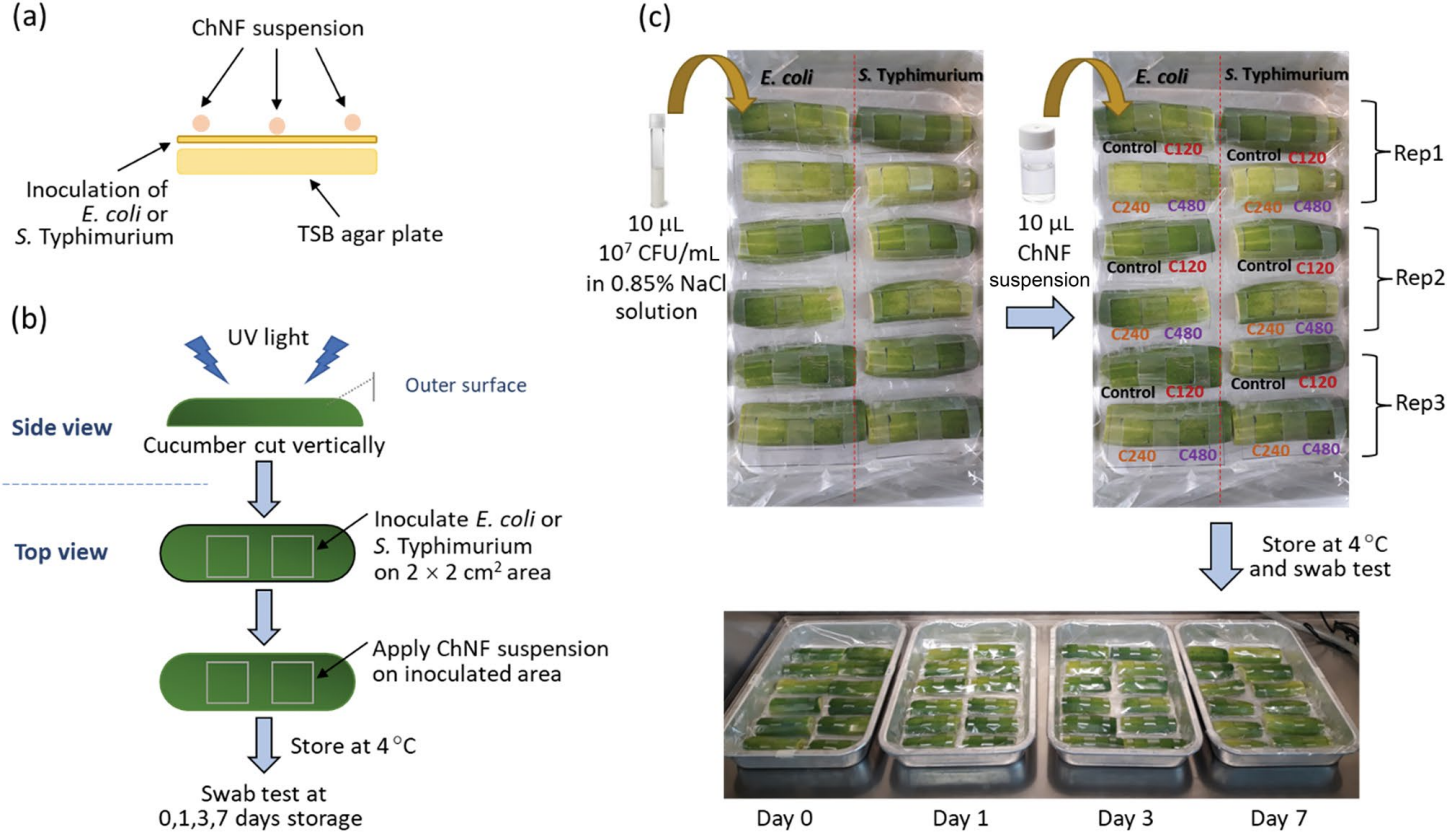
Schematic diagram of the bacterial challenge test. (**a**) Antimicrobial activity assay by spot-on-lawn method; (**b**) cucumber preparation and bacterial challenging; (**c**) layout of cucumber treatments.

The in vitro antimicrobial activities of the ChNF samples against *Escherichia coli* (*E. coli* ATCC 25922) and *Salmonella* Typhimurium (*S.* Typhimurium ATCC 13311) were determined using the spot-on-lawn method^42^ (Fig. 1a). The experiment was conducted in aseptic conditions. Each bacterium was inoculated separately in tryptic soy broth (TSB) and incubated at 37 °C for 24 h. A 50-µL sample of each culture was then mixed with 5 mL molten TSB soft agar (TSB + 1% agar) and poured onto a TSB agar plate. Once the agar plate was set, 10 µL of the ChNF suspension (0.75 wt%) was spotted on the agar surface (i.e., indicator lawn). The control was prepared by replacing the ChNF suspension with 1% acetic acid. The plate was then dried under sterile conditions and incubated at 37 °C for 24 h. A clear zone around the spot indicated bacterial inhibition.

In the second experiment, the antibacterial activities of the ChNF suspensions against *E. coli* and *S.* Typhimurium on cucumber outer surfaces during storage were determined (Fig. 1b). All steps were conducted under aseptic conditions. Fresh cucumbers were cut vertically in half and arranged on sterile aluminum trays. The cucumber’s outer surfaces were ultraviolet (UV)-sterilized for 15 min in a biosafety cabinet. A 2 × 2 cm^2^ area of the sterilized cucumber’s outer surface was inoculated with 10 µL of *E. coli* or *S.* Typhimurium suspension (7 log CFU mL^−1^ in sterilized 0.85% NaCl solution) and air-dried in a biosafety cabinet for 10 min. A 10-µL aliquot of the ChNF suspension (0.75 wt%) was then applied to the inoculated area and dried at room temperature under sterile conditions (Fig. 1c). For the control, the ChNF suspension was replaced with an equal volume of 1% acetic acid. The trays were then covered with a PE plastic bag and stored at 4 °C. The viability of *E. coli* and *S.* Typhimurium on the cucumber outer surfaces was monitored at 0, 1, 3, and 7 days of storage using a swab test. Briefly, a sterile cotton swab was rubbed against the inoculated cucumber outer surface and placed into 10 mL sterile normal saline (0.85% NaCl). Serial dilution was subsequently performed. *E. coli* and *S.* Typhimurium (expressed as log CFU cm^−2^) were enumerated on Chromocult Coliform agar (Merck, Germany) and xylose lysine deoxycholate agar (BD, USA), respectively. The experiments were performed in triplicate.

### Statistical analysis

Statistical analysis was performed using the R package (R.4.1.1). The statistical significance was set at α = 0.05. The mean differences were subsequently examined using Duncan’s multiple range test. For the bacterial challenge test on the cucumbers, the data and logarithmic-transformed data did not follow a normal distribution. Thus, the bacterial challenge test data were analyzed using a nonparametric Kruskal–Wallis method followed by Dunn’s nonparametric all-pairs comparison.

## Results and discussion

### Chemical structure

**Figure 2:**
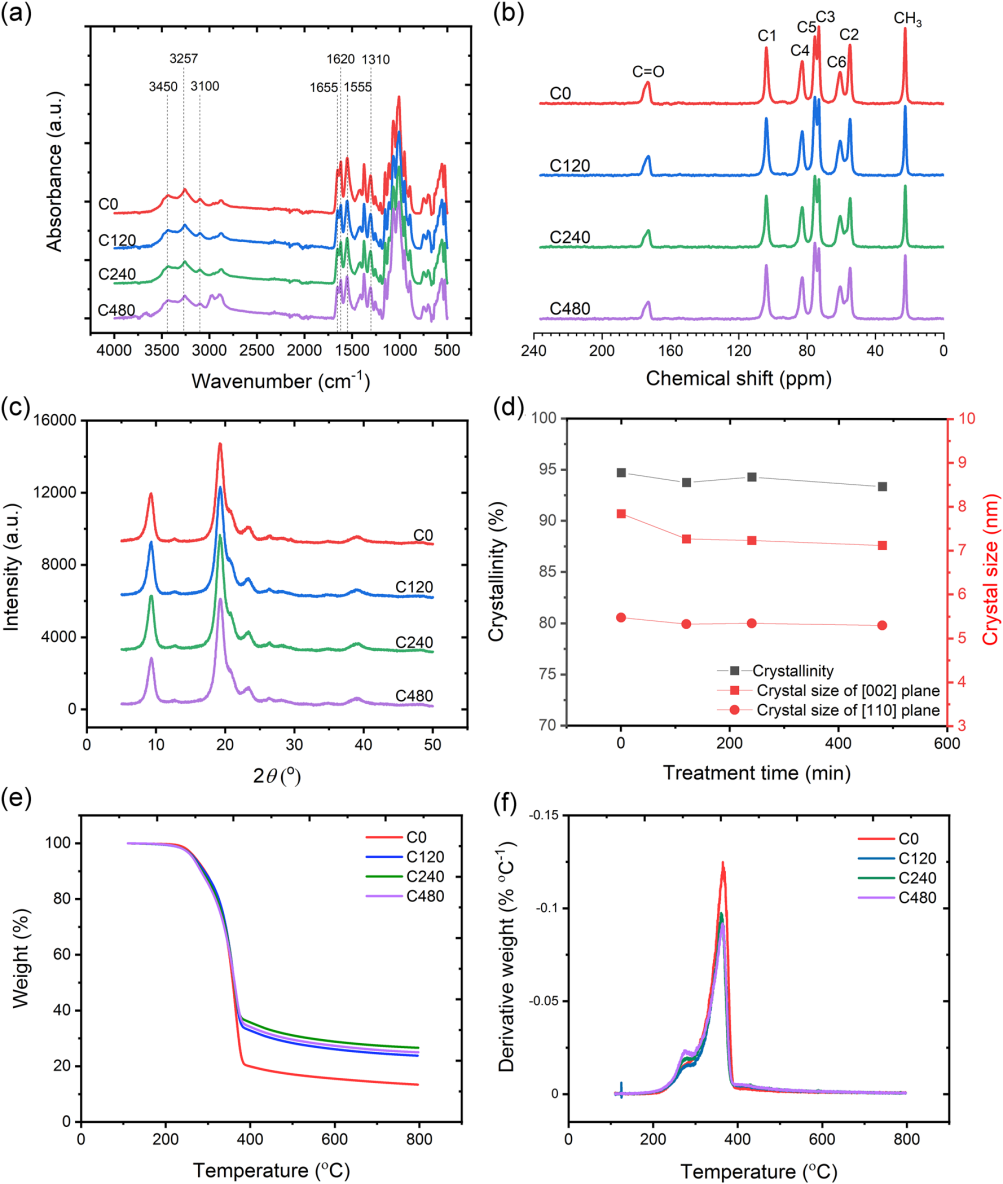
(**a**) Fourier-transform infrared (FTIR) and (**b**) ^13^C nuclear magnetic resonance (NMR) spectra of chitin nanofibers (ChNFs) with different deacetylation treatment times. (**c**) X-ray diffraction (XRD) patterns, (**d**) crystallinity and crystal sizes, and (**e**) thermogravimetric (TG) and (**f**) derivative thermogravimetric (DTG) curves of ChNFs as a function of the deacetylation treatment time.

Successful deacetylation of ChNFs using NaOH with various treatment times was evaluated using FTIR (Fig. 2a). The peak corresponding to intra- and intermolecular OH stretching occurred at 3450 cm^−1^, and NH stretching and NH secondary amide vibrations were observed from the peaks located at 3257 and 3100 cm^−1^, respectively^8,10,43,44^. Furthermore, doublets attributed to amide I (C=O stretching) were found at 1655 and 1620 cm^−1^, and the amide II band (N–H bending) at 1555 cm^−1^ and amide III band (C–N stretching) at 1310 cm^−1^ were observed^19,43–45^. Notably, the absorption band related to the existence of protein (⁓ 1420 cm^−1^) was not observed in all ChNF materials, suggesting that the multiple chemical treatment steps used herein could remove proteins and purify chitin particles^10,19,44^. Deacetylated ChNF samples (C120, C240, and C480) presented a less intense absorption band at 1655 cm^−1^ (related to the amide structure) than the non-deacetylated ChNF sample (C0). With increasing deacetylation treatment time, the intensity of this amide band decreased gradually, which was attributed to the conversion of acetamido groups in chitin molecules to amino groups. Notably, the amino band at ⁓ 1588 cm^−1^ was not observed for the ChNFs owing to the strong domination of the amide I and II bands over the amino bands^10,46^. However, with lower DA values, the amino band shifted toward a higher wavenumber due to reduced overlapping of the amide bands^46^.

Table 1 presents the DAs of the ChNFs as a function of the deacetylation treatment time. The main resonance peaks of the chitin nanoparticles were located at 173.4 (C=O), 103.9 (C_1_), 82.9 (C_4_), 75.5 (C_5_), 73.2 (C_3_), 60.7 (C_6_), 54.9 (C_2_), and 22.6 ppm (CH_3_) (Fig. 2b). The DA of C0 was 95.3%. After treatment with 30% NaOH for 120 min, the DA of C120 considerably reduced to 76.2%, i.e., ⁓ 25% of acetamido groups were converted to amino groups. Gradual conversion of acetamido groups to amino groups was found with increasing treatment time. Reductions in DA to 73.0% and 69.2% were observed for C240 and C480, respectively. The greater conversion during the initial deacetylation time was attributed to the deacetylation process, which primarily interacted with the chitin surface before penetrating the interior of the chitin fibril bundles^32^.

**Table 1:** Degree of acetylation (DA), degree of crystallinity, crystal sizes, temperature at 1% weight loss (*T*_1%_), and maximum degradation temperature (*T*_max_) of the chitin nanofibers (ChNFs) as a function of the deacetylation treatment time.

Property	C0	C120	C240	C480
DA (%)	95.3	76.2	73.0	69.2
Degree of crystallinity (%)	94.1	93.8	94.3	93.4
Crystal size of [020] plane (nm)	7.8	7.3	7.2	7.1
Crystal size of [110] plane (nm)	5.5	5.3	5.3	5.3
*T*_1%_ (°C)	237.0	225.3	222.6	220.2
*T*_max_ (°C)	363.6	359.5	358.8	359.3

### Crystallinity

Figure 2c shows the XRD profiles and crystalline indexes of the deacetylated ChNFs. All ChNF samples had five typical diffraction peaks at 9.3°, 19.4°, 21.2°, 23.4°, and 26.5°, attributed to the [020], [110], [120], [130], and [013] lattice planes, respectively^19,47^. These characteristic diffraction peaks were in agreement with those of the *α*-chitin structure^20,27,29^. The crystallinity of C0 was 94.1%. Notably, the crystallinity index of chitin depends on the raw materials and chemical treatment steps used in its purification^48,49^. Furthermore, the effect of deacetylation treatment time on the degree of crystallinity of chitin was evaluated. Deacetylation time had no considerable influence on the crystallinity of the ChNF materials (Fig. 2d; Table 1), which might be because deacetylation only occurred on the chitin crystallite surface when low NaOH concentration was used^20,27^.

Furthermore, the crystal sizes of the [020] and [110] planes of the ChNF samples as a function of the deacetylation time, calculated from the XRD profiles, are presented in Fig. 2d. C0 had crystal sizes in the [020] and [110] planes of 7.8 and 5.5 nm, respectively. Both crystal sizes in the [020] and [110] planes decreased with increasing deacetylation treatment time. The crystal sizes in the [020] and [110] planes of C240 were 7.2 and 5.3 nm, respectively, whereas the crystal sizes in the [020] and [110] planes of C480 were 7.1 and 5.3 nm, respectively. This indicated that the deacetylation reaction with longer processing time could yield ChNFs with smaller widths in the range of 5–7 nm.

### Thermal stability

The thermal stabilities of the deacetylated ChNFs were investigated with respect to treatment time. The TG and derivative thermogravimetric (DTG) curves of the deacetylated ChNFs as a function of treatment time are presented in Fig. 2e and f. Typically, the initial thermal transition state occurs at < 100 °C due to the evaporation of water bound to chitin by hydroxyl and amino groups^33,50^. However, this transition stage was not found in the TG curves of the ChNF samples as all ChNF samples were held at 110 °C for 20 min to completely remove water adsorbed or hydrogen-bonded with the chitin molecules^50,51^. The majority of chitin thermal degradation occurs from 200 to 400 °C, attributed to chitin-chain depolymerization associated with the thermal decomposition of pyranose rings via cleavage of the glycosidic linkages between *N*-glucosamine and *N*-acetyl glucosamine rings^50,51^. C0 presented a single DTG peak with a maximum degradation temperature (*T*_max_) of 363 °C, whereas the deacetylated ChNFs showed the main degradation temperature region at ⁓ 359 °C with a small crest at ⁓ 320 °C. The appearance of the thermal degradation peak at ⁓ 320 °C was attributed to the degradation of 2-amino-2-deoxy-d-glucopyranose units^52,53^. With increasing deacetylation time, the peak located at ⁓ 320 °C became more pronounced, but the intensity of the second peak at ⁓ 359 °C reduced. This was due to the high amount of amino groups in chitin molecules, which are less thermally stable than acetamido groups^54^.

Moreover, the thermal stability of the deacetylated ChNF samples decreased with increasing deacetylation treatment time owing to the high content of amino groups in the chitin molecules (Table 1). The thermal degradation temperature at 1% weight loss (*T*_1%_) of C0 was 237.0 °C. After deacetylation treatment for 120 min, the *T*_1%_ of C120 was considerably reduced to 225.3 °C. The reduced thermal degradation temperature of C120 was attributable to the partial conversion of acetamido groups to amino groups. Likewise, C240 and C480 exhibited *T*_1%_ values of 222.6 °C and 220.2 °C, respectively. Additionally, a reduction in the *T*_max_ of ChNFs occurred with the introduction of deacetylation. The *T*_max_ values of C0 and C120 were 363.6 °C and 359.5 °C, respectively. However, no significant change in *T*_max_ was observed with increasing deacetylation processing time. Additionally, a higher content of char residues at 800 °C (> 24.0%) was observed in the deacetylated ChNFs, compared with the non-deacetylated ChNFs (C0) (13.5%). This occurred because of the higher amount of amino groups available in the deacetylated chitin structures^55^. Therefore, a focus of our future work would be the application of deacetylated ChNFs as a reinforcing agent in polymeric matrixes for enhanced mechanical and flame-retardant properties^55,56^*.*

### Suspension stability and surface charges

**Figure 3:**
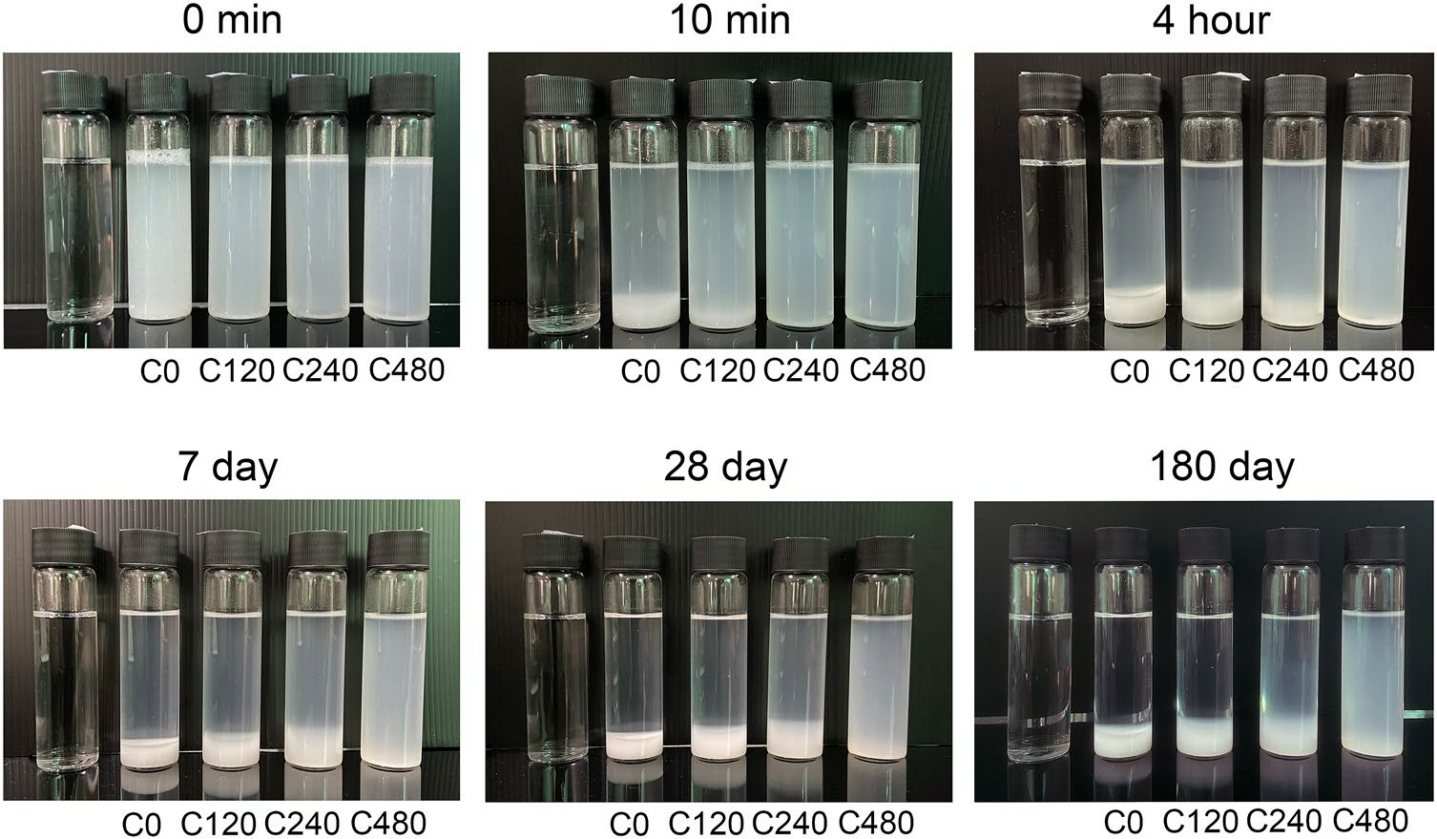
Photographs of the deacetylated ChNFs dispersed in distilled water with a few drops of acetic acid (pH ⁓ 3) as a function of precipitation time: immediately after blending (0 min) and after storing at room temperature for different times compared with distilled water (left).

The effect of deacetylation treatment time on the suspension stability of ChNFs was studied. Figure 3 presents the stability of the ChNF suspensions with different storage times. The C0 suspension (without deacetylation) showed rapid precipitation after 10 min, whereas noticeable sedimentation of C120 was observed after storing it at room temperature for 4 h. Clear precipitation of C240 was observed after 7 days, but flocculation was not observed for the C480 suspension even after 180 days. This indicated greater stability of the ChNF suspension with longer deacetylation treatment time. The superior stability of C480 was attributed to the high conversion of acetamido groups to amino groups in the chitin structure. Due to the protonation effect of these amino groups (NH_3_^+^), higher individualized nanofibers were fibrillated and reduced aggregation occurred via electrostatic repulsive forces^10^. Li et al*.*^10^ compared non-deacetylated and deacetylated ChNFs extracted from the same chitin source and found that although the geometric architectures of the non-deacetylated and deacetylated ChNFs were not affected by deacetylation treatment, the higher content of amino groups in the deacetylated ChNF structure could reduce fiber aggregation, resulting in higher stability of the deacetylated ChNF suspension. Moreover, a decrease in viscosity by one order of magnitude was observed after deacetylation treatment.

The positive charges on the ChNF surfaces were investigated with respect to the deacetylation reaction time via ζ-potential analysis. The average ζ-potential value of ChNFs was found to increase with increasing deacetylation treatment time. C0 had a ζ-potential value of + 20.3 mV, and after deacetylation for 120 min, the ζ-potential considerably increased to + 31.9 mV (C120). The ζ-potential values of + 42.0 and + 44.0 mV were measured for C240 and C480, respectively. The increase in ζ-potential supported the higher conversion of acetamido groups on the chitin backbone to amino groups with longer deacetylation treatment time^21^.

### Morphology

**Figure 4:**
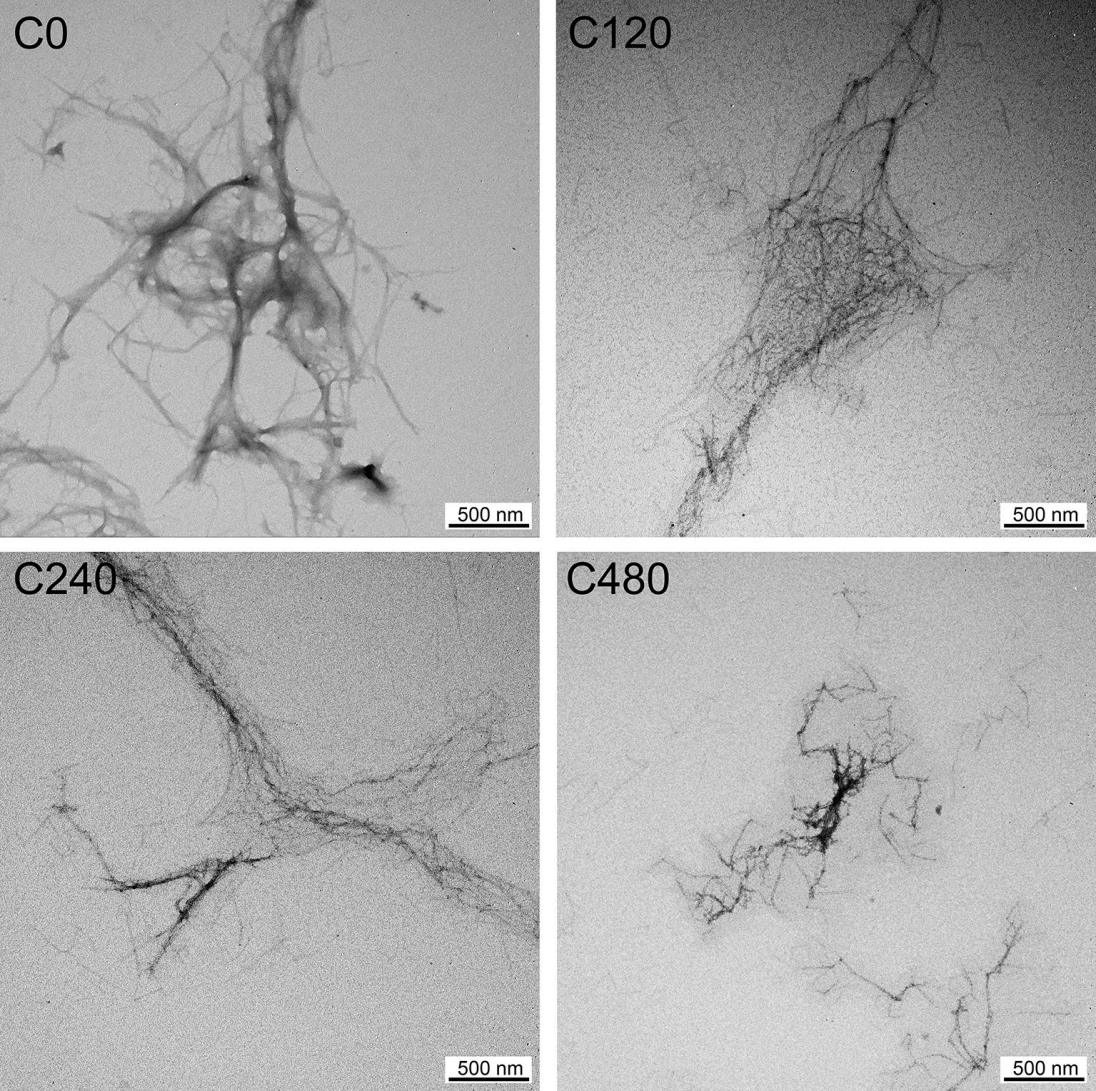
Transmission electron microscopy (TEM) images of the deacetylated ChNFs.

TEM images of the ChNFs after various deacetylation treatment times (0–480 min) are compared in Fig. 4. The defibrillation of nanofibers from shrimp exoskeletons was conducted for all chitin materials with or without deacetylation treatment. C0 with widths of < 20 nm disintegrated and large nanofiber aggregates in the range of 100–300 nm were observed. The presence of these fiber bundles was caused by strong hydrogen bonding between nanofibers associated with a high degree of crystallinity^27,30^, which caused the rapid deposition of C0 after 10 min (Fig. 3). However, the acetamido groups were converted to amino groups after deacetylation, reducing the intermolecular bonding between nanofibers^27^. This led to individualization of the ChNFs via simple mechanical disintegration^27,30^. For C120, the number of individualized ChNFs with widths of < 20 nm increased; however, large bundles of fibers were still present. Successful fibrillation of individualized spaghetti-like ChNFs occurred after the chitin particles were deacetylated for 240 min; C240 had an average width of 9.0 ± 1.8 nm and lengths of up to several μm. This was attributed to the high electrostatic repulsive forces resulting from the protonation of amino groups on the chitin structure between fibers, as confirmed by ζ-potential and NMR analyses^25,30^. Notably, during disintegration by high-speed blending, the pH of the chitin dispersion was reduced to ⁓ 3 using acetic acid, which generated positive charges on the amino groups (NH_3_^+^)^25,30^. The widths of ChNFs prepared herein were similar to those of ChNFs prepared via industrial high-pressure homogenization and grinding^10,25^. However, the morphological architecture of C480 unexpectedly changed from a fibril-like structure to a rod-like structure. The average width and length of C480 were 7.3 ± 1.8 nm and 222.3 ± 94.4 nm, respectively. The shortening of the nanofibers might result from the combination of the long alkaline deacetylation treatment and mechanical fibrillation. Ji et al*.*^57^ discovered that the deacetylation predominantly interacted with the amorphous region of chitin, leading to the dissolution of the deacetylated amorphous part in an acidic solution. Consequently, this resulted in the shortened length of ChNFs^58^. A similar phenomenon of the length reduction was observed for ChNFs with a lower DA. As the DA of ChNFs decreased from 89.2 to 71.6%, the average length of ChNFs exhibited a significant decrease from 895 ± 551 nm to 428 ± 270 nm^58^.

### Shelf life extension of cucumbers

**Figure 5:**
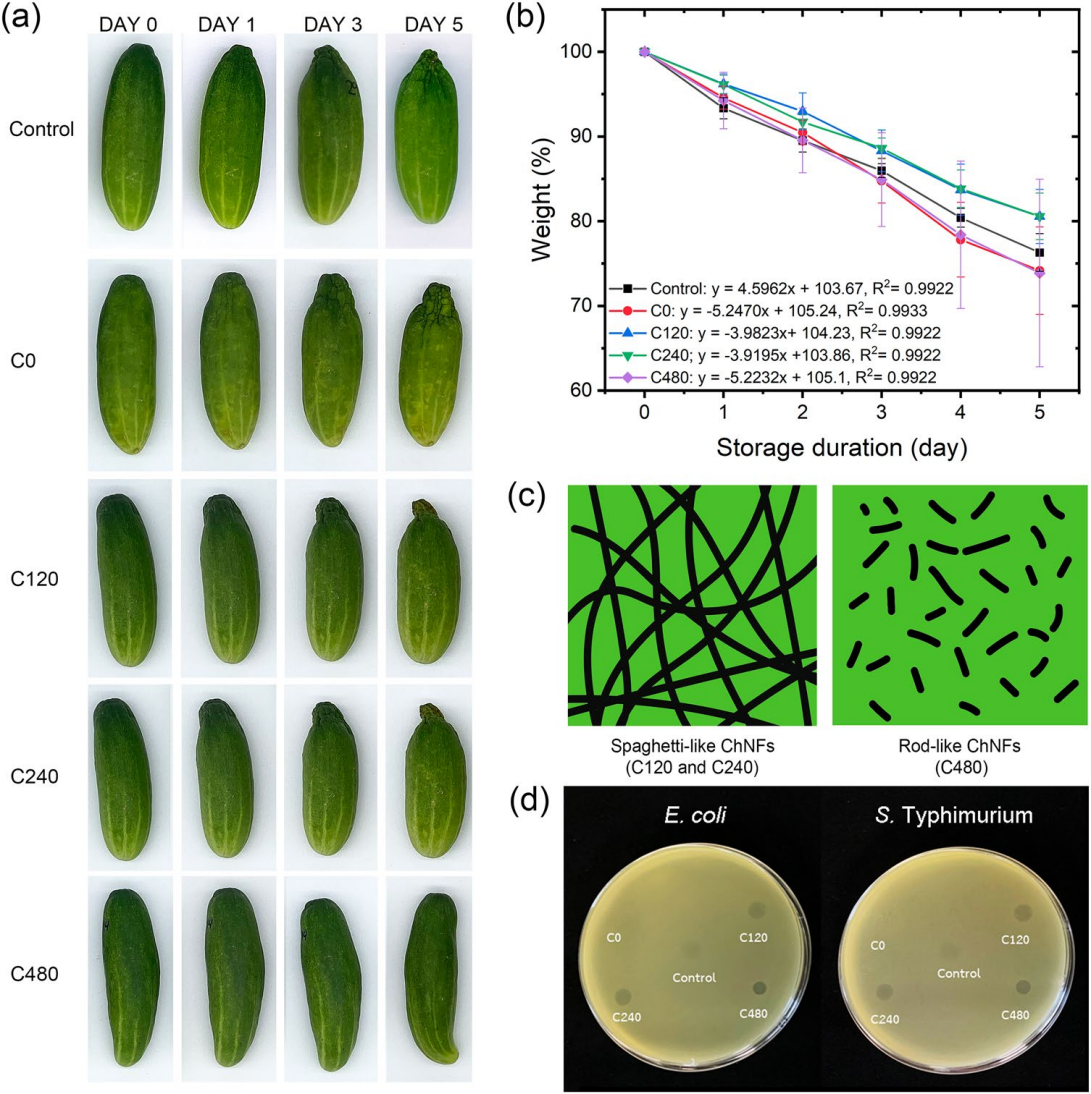
(**a**) Visual appearance and (**b**) weight change of cucumbers coated with ChNFs prepared with various deacetylation times (control, C0, C120, C240, and C480) conditioned at 30 ± 3 °C as a function of storage time. (**c**) Schematic illustrating spaghetti-like structured ChNFs (C120 and C240) and rod-like structured ChNFs (C480) covered on cucumber surfaces, and (**d**) clear zones of inhibition of ChNFs prepared with various deacetylation times (C0, C120, C240, and C480) against *Escherichia coli* (*E. coli*) and *Salmonella* Typhimurium (*S.* Typhimurium). 1% acetic acid was used as control.

Herein, the application of ChNF suspensions for the shelf life extension of fresh cucumbers was investigated. Fresh cucumbers were selected as a food model to test the efficiency of the ChNF coatings as they are an economically important fresh product consumed worldwide. Local cucumbers were directly coated with the ChNF suspensions with the concentration of 0.75 wt% (C0, C120, C240, and C480) and monitored daily. The visual appearance of the coated and uncoated cucumbers is presented in Fig. 5a, and the weight loss of the cucumbers as a function of storage time is presented in Table 2. The freshness of fruits and vegetables can be determined by color change and weight loss^59,60^. Weight loss is a crucial factor, which implies the quality and shelf life of crop products^61,62^. Water or moisture loss from fresh produce could lead to metabolic changes in plant cells, which accelerates senescence and negatively affects the nutritional content of the produce^61^. After 1 day of storage, all cucumber samples remained green and fresh with no signs of decay. No difference in weight loss of the cucumbers was observed with the application of the ChNF coatings between days 0 and 1. However, the uncoated cucumber (control) showed the significant weight loss between days 0 and 1 (*p* < 0.05). This weight loss was primarily attributed to moisture loss from the cucumbers^4,11^. With increasing storage duration, the weight loss of the cucumber samples increased for all treatments. After 3 days of storage, all cucumbers with and without ChNF coatings were still green, but an area close to the stalk was shriveled on all the samples. This agreed well with the weight loss results. This dryness was more pronounced on day 5 and was easily observed by the naked eye. The C120- and C240-coated cucumbers showed lower weight loss than the other samples (control, C0, and C480), whereas the cucumbers coated with C0 and C480 showed similar weight loss results to that of the uncoated cucumbers (control). This indicates that the C120 and C240 coatings could delay the moisture loss of cucumbers. The weight loss of the cucumbers was found to have a strong relationship with their volume loss^63^. Additionally, greater weight loss rates were observed as a function of storage duration in the cucumbers coated with C0 (− 5.25% day^−1^) and C480 (− 5.22% day^−1^) and the control samples (− 4.60% day^−1^) compared with that of the cucumbers coated with C120 (− 3.98% day^−1^) and C240 (− 3.92% day^−1^) (Fig. 5b). With increasing storage time, the difference between these two sets became more pronounced. The lower moisture release rate of the C120- and C240-coated cucumbers might be due to the greater fibrillation of their ChNFs (less aggregation) compared to C0 and the longer fiber lengths compared to C480. Fibers with smaller widths can form a network with smaller pore sizes^59,64^. Furthermore, adjacent long nanofibers would form an interconnected network with larger amounts of hydrogen bonding on the cucumber surfaces, which would retain water molecules^65^; however, the rod-like structured ChNFs might encounter challenges in forming a comparable network due to their shorter length, as shown in Fig. 5c.

**Table 2:** Weight change of cucumbers coated with ChNFs prepared with various deacetylation times with respect to storage duration.

Storage duration (day)	Weight of cucumbers coated with ChNFs (%)				
	Control	C0	C120	C240	C480
0	100.0 ± 0.0^a^	100.0 ± 0.0^a^	100.0 ± 0.0^a^	100.0 ± 0.0^a^	100.0 ± 0.0^a^
1	93.33 ± 1.26^bc^	94.06 ± 1.06^ab^	96.20 ± 1.19^ab^	96.14 ± 1.09^ab^	94.23 ± 3.31^ab^
3	85.96 ± 0.81^de^	87.75 ± 2.61^de^	88.30 ± 2.47^cd^	88.62 ± 1.19^cd^	84.90 ± 5.53^de^
5	76.30 ± 2.23^fg^	74.16 ± 5.16^g^	80.55 ± 3.21^ef^	80.57 ± 2.76^ef^	73.87 ± 11.06^g^

Different superscripts indicate statistical differences (*p* < 0.05) among samples.

Additionally, compared with short nanofibers, longer nanofibers would form a longer diffusion pathway on cucumber surfaces that would delay the transportation of gas molecules^66^. The permeation of gas molecules such as water and O_2_ through pores within a nanofiber network would also be controlled by the network density^65^. These findings suggested that the ChNFs (C120 and C240) used as coatings to extend the shelf life of fresh cucumbers could potentially be applied to other fresh fruits and vegetables, enabling a reduction in single-use packaging, which damages our environment and endangers both humans and animals.

### Antibacterial activity and bacterial inhibition on cucumbers

Qualitative evaluation of the antimicrobial activity of ChNFs was performed against *E. coli* and *S.* Typhimurium, two Gram-negative foodborne pathogens generally found in raw products from both plant and animal origins^67–69^. A sharp clear zone of inhibition against *E. coli* and *S.* Typhimurium was observed when 10 µL of C120, C240, or C480 solution (0.75 wt%) were directly dropped onto the bacterial lawn, whereas this was not observed for C0 (Fig. 5d). The results suggested that deacetylation time and DA affected the antimicrobial activity of ChNFs against the tested bacteria. Similar tendency was observed by Tsigos et al.^70^, Hongpattarakere and Riyaphan^71^, and Benhabiles et al*.*^72^, who reported that the antimicrobial activity of chitin and chitosan was partly improved by the deacetylation process. Herein, deacetylation for 120 min was sufficient to convert inactive ChNFs (C0, DA = 95.3%) to antimicrobial ChNFs (C120, DA = 76.2%).

Based on these antibacterial activities observed in vitro using the spot-on-lawn method, only C120, C240, and C480 were tested against *E. coli* and *S.* Typhimurium on fresh cucumber outer surfaces. This experiment aimed at investigating the antimicrobial effects of ChNFs under intended-use condition on the surface of fresh produce. The bacterial challenge study revealed the antimicrobial efficiency of the tested ChNFs against both *E. coli* and *S.* Typhimurium on cucumbers during chilled storage (Table 3). All tested ChNFs effectively inhibited the growth of *E. coli* by maintaining bacterial viability at the inoculated levels throughout 7 days of storage; however, the number of *E. coli* in the control group significantly increased from the first day of storage. No significant differences (*p* > 0.05) were observed among the ChNF groups during this storage period. The results suggested that the potencies of C120, C240, and C480 against *E. coli* on this food model were not different. As for *S.* Typhimurium, applications of C120 and C240 on the cucumber outer surfaces significantly decreased (~ 90%) viability of the bacteria within a day. The results indicated that *S.* Typhimurium was rapidly killed when exposed to C120 and C240. However, on day 3 of storage, the *S.* Typhimurium numbers in the C120 and C240 groups increased to their initial level and were not different from that of the control (*p* > 0.05). This result suggested that the applied concentration of C120 and C240 might be insufficient to kill all bacteria on the cucumber surfaces; thus, residual viable cells were able to grow afterwards. Conversely, as for C480, the viability of *S.* Typhimurium on the cucumber surfaces did not significantly change during the storage, although its antimicrobial activity was visualized by the spot-on-lawn assay. The findings suggested that, unlike C120 and C240, C480 might not kill the bacteria. Instead, it might exhibit bacteriostatic (bacterial inhibition) action against *S.* Typhimurium.

**Table 3:** Changes in bacterial viability on cucumber outer surfaces with different ChNF coatings (C120, C240, and C480) during storage at 4 °C.

Bacteria	Storage duration (day)	Bacterial viability on cucumber outer surfaces (log CFU cm^−2^)			
		Control	C120	C240	C480
*E. coli*	0	4.42 ± 0.23^Ab^	4.62 ± 0.00^Aa^	4.42 ± 0.10^Aa^	4.52 ± 0.28^Aa^
	1	5.72 ± 0.08^Aa^	5.35 ± 0.39^ABa^	4.63 ± 0.15^Ba^	4.47 ± 0.05^Ba^
	3	5.62 ± 0.01^Aa^	5.10 ± 0.18^Aa^	4.93 ± 0.11^Aa^	5.47 ± 0.39^Aa^
	7	5.82 ± 0.12^Aa^	5.29 ± 0.18^ABa^	5.06 ± 0.46^Ba^	5.31 ± 0.05^ABa^
*S.* Typhimurium	0	4.57 ± 0.20^Aa^	4.34 ± 0.05^Aa^	4.36 ± 0.01^Aa^	4.56 ± 0.01^Aa^
	1	4.51 ± 0.00^Aa^	3.26 ± 0.04^Bb^	3.49 ± 0.05^Bb^	4.51 ± 0.01^Aa^
	3	4.37 ± 0.17^Aa^	4.32 ± 0.02^Aa^	4.18 ± 0.13^Aa^	4.38 ± 0.00^Aa^
	7	4.63 ± 0.00^Aa^	4.58 ± 0.03^ABa^	4.30 ± 0.17^Ba^	4.51 ± 0.00^Aa^

Control groups were inoculated on cucumber surfaces with a 1% acetic acid coating. Different capital letters in the same row indicate significant differences (*p* < 0.05) among treatments on the same storage day. Different lowercase letters in the same column indicate significant differences (*p* < 0.05) among storage days within the same treatment.

The precise antimicrobial mechanism and factors affecting the antimicrobial activity of chitin are still underway. The mechanism most often proposed is the electrostatic stacking of positively charged chitin on the negatively charged bacterial cell surface, which consequently interferes with the cellular metabolism and cell membrane permeability of the bacteria^10,73,74^. Xu et al*.*^75^ found that the antibacterial properties of ChNFs was strongly dependent on the DA due to the higher amounts of amino groups. In this regard, it was initially expected that C480 would inhibit the tested bacteria to a greater extent than C120 and C240. Indeed, the discrepancy requires further investigation. However, the findings suggested that, rather than the charge alone, there are other factors (e.g., fiber width and length) affecting antimicrobial activities of the ChNF. Herein, C120, C240, and C480 had slight differences in DA (from 76 to 69%) and ζ-potential (from + 31.9 to + 44.0 mV). It could be speculated that shorter ChNFs (a rod-like structure) might be insufficient to stack or cover the bacterial cell surface, thus exhibiting weaker antimicrobial action (Fig. 5c). This was agreed with the higher weight loss rate of the cucumbers coated with C480 than the cucumbers coated with C120 and C240.

Overall, the current findings suggested that the ChNF (C120 and C240) suspensions can extend the shelf life of fresh cucumbers by reducing moisture loss and delaying the growth of *S.* Typhimurium, which linked thousands of cases of *Salmonella* spp. outbreaks in North America^76,77^ and the UK^78^. Furthermore, chitin, a biodegradable, biocompatible, and non-toxic material, has been widely used in various applications including biomaterials, food preservation, cosmetic, and pharmaceuticals^17,18,79^. However, concerns have been raised regarding potential seafood allergies triggered by chitin. Seafood allergies, particularly shrimp allergy, have been associated with muscle proteins found in shellfish^80^. It is important to note that proteins were removed during the preparation process of chitin. A study of the safety of chitosan (a derivative of chitin) in individuals allergic to shrimp found that wine processed with chitosan as a preservative agent did not induce allergic reactions in patients with shrimp allergy^80^. Since chitin and chitosan have similar structures, this research provides additional insight into the utilization of ChNFs and supports the safety of their consumption. In our future work, we plan to explore the residue of ChNFs on fresh produce after washing, which will help us understand the potential limitation of the ChNF coatings.

## Conclusion

ChNFs with various DAs (95%, 76%, 73%, and 69%) were successfully developed from shrimp shell waste via deacetylation using NaOH and mechanical defibrillation. Longer deacetylation reaction times resulted in greater replacement of acetamido groups by amino groups in chitin molecules, reducing DA and thermal stability. These amino groups imparted promising antibacterial activity to the ChNFs. With increased reaction time, the individualization of ChNFs and higher stability of the ChNF suspension were realized. Additionally, the spaghetti-like structure of ChNFs was converted to a rod-like structure when the deacetylation reaction time increased to 480 min. Furthermore, we applied these ChNFs as a coating to extend the shelf life of fresh cucumbers. The spaghetti-like ChNF suspensions (C120 and C240) considerably prevented moisture loss and delayed the growth of *S.* Typhimurium on the cucumber outer surfaces compared with the individualized rod-like C480. Therefore, sustainable ChNF suspensions are a promising approach to reducing food waste and replacing petroleum-based polymers for food packaging.

## Data Availability

The datasets generated and analyzed during the current study are available from the corresponding author on reasonable request.
